# Bed net ownership in Kenya: the impact of 3.4 million free bed nets

**DOI:** 10.1186/1475-2875-9-183

**Published:** 2010-06-24

**Authors:** Allen Hightower, Rebecca Kiptui, Ayub Manya, Adam Wolkon, Jodi Leigh Vanden Eng, Mary Hamel, Abdisalan Noor, Shahnaz K Sharif, Robert Buluma, John Vulule, Kayla Laserson, Laurence Slutsker, Willis Akhwale

**Affiliations:** 1 Division of Parasitic Diseases and Malaria, Centers for Disease Control, Center for Global Health, Mailstop F22, 4770 Buford Highway, Atlanta GA 30341, USA; 2 Division of Malaria Control, Ministry of Public Health and Hygiene, KNH Grounds, P.O. Box 20750, Nairobi, Kenya; 3 Kenya Medical Research Institute, Center for Global Health Research, Off Busia Road, Kisian, Kenya; 4 Malaria Public Health and Epidemiology Group, Centre for Geographic Medicine Research-Coast, Kenya Medical Research Institute/Wellcome Trust Research Programme, P.O. Box 43640, 00100 GPO, Nairobi, Kenya; 5 Ministry of Health, Office of the Director of Public Health and Sanitation, Afya House, Cathedral Road, P.O. Box 30016, Nairobi, Kenya P.O. Box 30016, Nairobi; 6 Kenya National Bureau of Statistics, Herufi House, Lt. Tumbo Lane, P.O. Box 30266-00100 GPO, Nairobi, Kenya; 7 Center for Global Health, Centers for Disease Control, 1600 Clifton Road, Atlanta GA 30333, USA

## Abstract

**Background:**

In July and September 2006, 3.4 million long-lasting insecticide-treated bed nets (LLINs) were distributed free in a campaign targeting children 0-59 months old (CU5s) in the 46 districts with malaria in Kenya. A survey was conducted one month after the distribution to evaluate who received campaign LLINs, who owned insecticide-treated bed nets and other bed nets received through other channels, and how these nets were being used. The feasibility of a distribution strategy aimed at a high-risk target group to meet bed net ownership and usage targets is evaluated.

**Methods:**

A stratified, two-stage cluster survey sampled districts and enumeration areas with probability proportional to size. Handheld computers (PDAs) with attached global positioning systems (GPS) were used to develop the sampling frame, guide interviewers back to chosen households, and collect survey data.

**Results:**

In targeted areas, 67.5% (95% CI: 64.6, 70.3%) of all households with CU5s received campaign LLINs. Including previously owned nets, 74.4% (95% CI: 71.8, 77.0%) of all households with CU5s had an ITN. Over half of CU5s (51.7%, 95% CI: 48.8, 54.7%) slept under an ITN during the previous evening. Nearly forty percent (39.1%) of all households received a campaign net, elevating overall household ownership of ITNs to 50.7% (95% CI: 48.4, 52.9%).

**Conclusions:**

The campaign was successful in reaching the target population, families with CU5s, the risk group most vulnerable to malaria. Targeted distribution strategies will help Kenya approach indicator targets, but will need to be combined with other strategies to achieve desired population coverage levels.

## Background

The last, large-scale, group-randomized, controlled trial of insecticide-treated bed nets (ITNs) showed that ITNs were efficacious in reducing all-cause post-neonatal mortality in an area of Western Kenya with intense, perennial malaria transmission [1-3]. That trial and others [4-7] helped to define pregnant women and children 0-59 months old (CU5s) as target groups for ITNs in high transmission settings. The findings also suggested that high overall population coverage with ITNs including both target and non-target groups was critical to achieve community level protective effects [3]. These studies emphasized the beneficial effects of high ITN coverage from both health and economic perspectives.

Key determinants of efficacy in this and other ITN studies were the proportion of households with ITNs (ownership), the proportion of individuals properly deploying ITNs each night (usage), and the proportion of nets properly treated with insecticide (treatment). These indicators have been adapted for programme monitoring and evaluation. Indicators for ITN ownership and usage are an integral part of a number of development goals, including those set forth in the Abuja Declaration [8], Roll Back Malaria Strategic Document (RBM) [9], and the Millennium Development Goals [10]. The 2010 RBM targets are: 80% of CU5s sleeping under an ITN during the previous night, and 80% of pregnant women sleeping under an ITN during the previous night. In the past few years, long-lasting ITNs (LLINs) have become widely available; these are nets that are treated during the manufacturing process and are protective for an estimated three years.

One method to rapidly achieve high coverage is a large-scale integrated campaign, where ITN distribution is linked to other child health interventions, such as immunizations or Vitamin A supplementation [11,12]. One of the largest of these campaigns, involving 3.4 million LLINs, was conducted in Kenya in 2006. The outcomes of this campaign, retention of campaign LLINs, and estimates of the indicators to assess Kenya's progress towards goals for ITN coverage at the household level and high-risk populations are reported here. Additionally, the survey provided a measure of the progress of ongoing and substantial efforts to distribute subsidized, socially marketed bed nets over the past four years in Kenya. The survey collected data using a novel approach that has been tested in other countries as being both statistically valid and rapid [13-16].

## Methods

### The campaign to distribute LLINs

1.4 million LLINs were distributed from July 8 to 14, 2006, primarily in Nyanza and Western provinces, in association with a measles vaccination campaign. Western and Nyanza provinces were chosen to receive nets first because of a high burden of malaria-related morbidity and mortality. The remaining 2.0 million nets were distributed September 23 to 24, 2006 in malaria-affected districts in Rift Valley, Central, Eastern, and Coast Provinces. The target population to receive the nets was children under five years of age. Batches of LLINs were sent to numerous distribution centers (health facilities, schools, etc.) where caretakers would receive one net for each CU5 that accompanied them. At some centers, the children received other health interventions appropriate for the locale (measles vaccination, deworming medication, vitamin A, and iron tablets).

### Survey design

There are 46 Districts in Kenya where malaria transmission occurs. Twenty of these Districts were randomly chosen with probability proportional to size within the following three malaria transmission zones that formed the sampling strata for the survey: areas of endemic malaria - 10 districts from Nyanza, Western, and Coast provinces (2006 estimated population: 11.9 million); epidemic malaria - five districts from the Rift Valley (2006 estimated population: 8.6 million); highland/seasonal malaria - five districts from Eastern and Central Provinces (2006 estimated population: 9.2 million). The region containing Nyanza, Western, and Coast Provinces had higher sampling fractions because they contained all but one of the endemic districts in the country and are where the majority of malaria-related morbidity and mortality occurs.

Within each district, five enumeration areas were selected with probability proportional to size. A sample of 21 households was selected for each enumeration area, giving a target sample of 2,100 households. The sampling frame within each cluster was developed through the use of global positioning system (GPS) linked with hand-held computers (PDAs) to allow rapid preliminary mapping of enumeration areas, thereby allowing selection of a simple random sample of houses [13]. Sufficient numbers of districts and enumeration areas were sampled in the Nyanza and Rift Valley provinces and the other two regions to produce statistically reliable estimates and confidence intervals.

### Data collection

**Figure 1:**
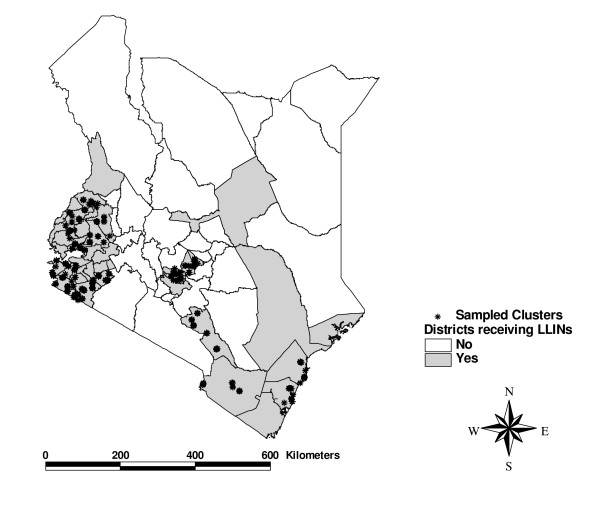
**Districts in the bed net distributions and clusters in the evaluation survey**.

The survey was conducted from October 17 to November 1, 2006 (Figure 1). Questions and methodology were similar to the 2003 Kenya Demographic and Health Survey (KDHS) to facilitate comparisons [17]. The survey instrument asked questions about the household, the bed nets owned by the household (if any), whether a CU5 or a woman of reproductive age had slept under each specific net during the previous evening, malaria knowledge and awareness of public media messages related to the campaign, and socio-economic questions concerning characteristics of the household. Surveyors noted whether they had actually observed the nets and if so, if they were hanging. Although pregnant women were not specifically targeted by the campaign, they were included in the survey to measure the effectiveness of the targeted campaign for CU5s in reaching them. Socioeconomic data used to construct a wealth index employing the same definitions used in the 2003 KDHS [17].

PDAs with GPS were used for data collection. The questionnaire was constructed and programmed into the PDAs with appropriate skip patterns and data checks. After a one-week training period, 11 teams of four persons each collected the data in the field over a two week period. Upon survey completion, PDAs were returned to Nairobi, where data were aggregated into a desktop database.

The survey was conducted at the end of the dry season and the early stage of the short rainy season which is not the peak time of malaria transmission. As a result it was not expected to provide the maximum measure of net usage. The objective of this survey was to assess ownership and retention of bed nets distributed during the campaign and to assess bed net ownership in general.

### Definitions

A household was defined as everyone who shared a meal at a common gathering place, using the 2003 Kenya DHS definition [17]. An ITN was defined as any bed net that had been treated with insecticide in the last six months. LLINs were counted as ITNs. At the time of the survey, there were only three types of LLINs available in Kenya: SupaNet ExtraPower (socially marketed by Population Services International), Permanets (available commercially and via the campaign), and Olyset nets (available commercially and via the campaign). Pre-campaign bed net ownership levels were based on ownership of non-campaign bed nets. Changes in ownership of these nets between the campaign and the survey were assumed to be negligible. Post-campaign bed net ownership included all types of bed nets. Permanet and Olyset campaign nets had distinctive labeling and packaging. To aid identification, the PDAs used for survey data collection had pictures of each net, and its packaging and labeling, integrated into the computerized questionnaire. Equity ratio was defined as the ratio of intervention coverage proportions in the poorest quintile to the coverage in the wealthiest quintile.

### Statistical methods

Consistent with the sampling design, the SAS survey procedures (SAS v9.1.3, SurveyFreq, SurveyLogistic, and SurveyMeans) were used to produce estimates and standard errors using the sampling weights that accounted for the sampling design and the different sampling fractions for each transmission zone. Estimates are precise (half-width of 95% confidence interval) to 2.5% on a national basis (i.e., for all areas with malaria in Kenya), 2.5-4.0% on a regional basis (the three sampling strata based on provinces grouped by the nature of their malaria endemicity), 5% for each wealth quintile, and 6% for CU5. The survey was not designed for making precise statements on bed net ownership or usage for pregnant women. However, precision for women of reproductive age was 5% on a national basis.

Comparisons of indicators before and after the campaign were done by computing the difference in the indicators and computing 95% confidence intervals that accounted for the survey design. The Rao Scott Chi square statistic was used for p-values. Projected numbers of various types of bed nets (ITNs, campaign nets) were computed by multiplying the number of a particular type of bed net in the survey household by its sampling weight and summing this product over all households.

### Ethical considerations

The protocol was approved by the Kenya Medical Research Institute Institutional Review Board and the Centers for Disease Control and Prevention. Informed written consent was obtained from each respondent.

## Results

### Respondents

From October 17 through November 1, 2006, members of 2,059 households were interviewed out of 2,100 targeted. Of the 100 enumeration areas, 85% were classified as rural and 15% as urban, mirroring the characteristics of the 46 campaign districts. On a weighted basis, households with CU5s represented 51.7% of all households in the survey population. Among sampled households, 78.0% had a woman of reproductive age; of these, 37.1% did not have a CU5 (95% CI: 34.7, 39.6%). An estimated 8.3% of the women in the population reported themselves to be pregnant.

### Before vs. after campaign comparisons for key indicators

**Figure 2:**
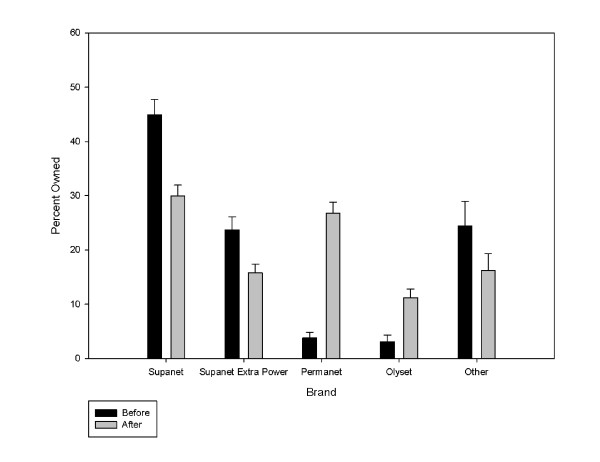
Distribution of bed net brands owned before and after the campaign*. * Pre-campaign: Number of bed nets = 2,105, Post-Campaign: Number of bed nets = 3,141.

The survey estimated that 3.63 million LLINs were distributed (95% CI: 3.39, 3.87 million), of which 3.38 million were distributed to CU5s (93.9%) (Table 1). The campaign increased the number of bed nets owned by 50%, from 7.2 to 10.9 million. Households owning only campaign nets were 13.8% of all households, which was over one-third of all households receiving campaign nets. Pre-campaign ITN household ownership could be estimated as 36.9% = 50.7 (post-campaign ITN/LLIN HH ownership) -13.8% (households that only owned campaign nets in the survey), and similarly pre-campaign household ownership of any bed net could be estimated at 53.1% (66.9-13.8%). Prior to the campaign, socially marketed nets represented 68.7% of all nets owned. This represents approximately 5.0 million nets (7.24 million* 0.687). After the campaign, they represented 46.0% of all nets owned. Figure 2 presents the distribution of bed net brand ownership before and after the campaign.

**Table 1:** Key indicators, before and after the campaign *

	Before campaign, (95% Confidence Interval)	After campaign, (95% Confidence Interval)
Total Number of Campaign Bed nets Distributed	none	3.63 million, (3.39, 3.87 million)

Total Bed nets Owned	7.24 million, (7.14, 7.34 million)	10.87 million, (10.53, 11.21 million)

Percent of All Bed nets that were Socially Marketed	68.7%, (66.0, 71.3)	45.7%, (43.5, 47.9)

HH with CU5 and ITN/LLIN	46.7%, (44.0, 49.4)	74.4%, (71.8, 77.0)

* Number of bed nets of all types in survey = 3141.

Number of ITNs (including LLINs) in survey = 2091, found in 1080 HH.

Number of HHs in survey = 2059

The campaign increased ITN ownership for HH with CU5s by 28.7% (from 46.7% to 74.4%, p < .0001). It increased ITN ownership for HH with women of reproductive age by 18.6% (from 40.3% to 58.9%).

Prior to the campaign, the percent of all nets in households with CU5s that were ITN increased with the wealth quintile. However, even in the wealthiest quintile, less than a third of the homes owned ITNs. After the campaign, the relationship between ITN ownership and wealth disappeared. The smallest increase in ITN ownership was in the wealthiest quintile, 25.5%,. The other wealth quintiles had increases of nearly 40% or more. The increases in all wealth quintiles were statistically significant (p < .001 for all quintiles, Table 2).

**Table 2:** Percent of all bed nets in households with children under 5 that were ITNs (including LLINs) by wealth quintile before and after the campaign.*

	Before campaign, (95% Confidence Interval)	After campaign, (95% Confidence Interval)	Change (95% Confidence Interval)
Poorest	19.9%, (16.0, 23.9)	59.1%, (53.7, 64.5)	39.2%, (34.2, 44.2)
Second Poorest	21.4%, (17.3, 25.5)	76.2%, (71.5, 80.9)	54.8%, (49.6, 60.0)
Mid Quintile	24.7%, (18.8, 30.7)	62.9%, (55.8, 70.1)	38.2% (31.4, 45.0)
Second Richest	27.6%, (22.6, 32.6)	66.1%, (60.5, 71.6)	38.5%, (33.0, 44.0)
Richest	30.6%, (25.4, 35.9)	56.1%, (50.1, 62.1)	25.5%, (20.5, 30.4)
Total	24.4%, (22.3, 26.6)	64.5%, (62.0, 67.1)	40.1%, (37.6, 42.6)

* Number of Households with CU5s in survey = 1113.

### Equity ratios

**Figure 3:**
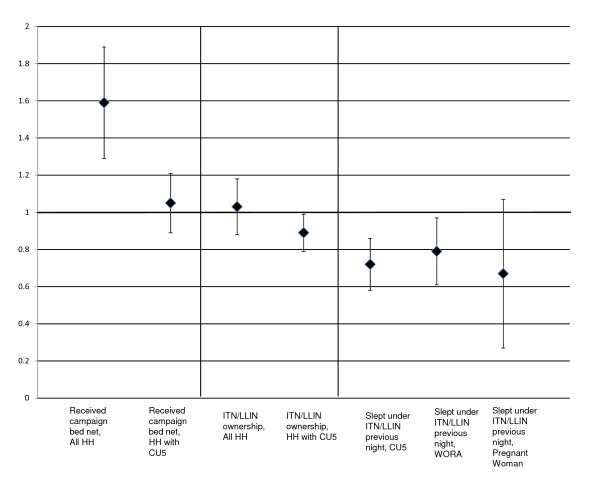
Equity ratios for selected indicators*. *An equity ratio of more than 1 means that the poorest quintile was more likely to receive the intervention, less than 1 means that the wealthiest quintile was more likely to receive the intervention. Sample sizes: Number of households: 2059; Number of Households with CU5s: 1113, Number of Households with Women of Reproductive Age (WORA): 1613; Number of CU5s: 1727, Number or WORAs: 2053, Number of Pregnant Women: 182.

The equity ratio for all households receiving campaign bed nets was 1.59 (95% CI: 1.29, 1.89). This means that the poorest households were 59% more likely to receive campaign bed nets than the wealthiest households. The equity ratio for CU5s, women of reproductive age, and pregnant women sleeping under an ITN the previous night were all less than 0.8. This means that despite the campaign, the target populations in the wealthiest quintiles were all 20% or more likely to be sleeping under an ITN. Equity ratios with 95% confidence intervals are presented in Figure 3.

### Bed nets

There were 3141 bed nets in the 2059 households in the survey. Over half of households surveyed (n = 1080) owned ITNs. One third of all nets were campaign nets. During the survey, 70% of all nets were seen by the interviewers. Summary data with 95% confidence intervals are presented in Table 3.

**Table 3:** Survey estimates related to bed nets*

	Percent, (95% Confidence Interval)
Bed nets that were either an ITN or LLIN	66.2%, (64.7, 69.1)

Bed nets that were LLINs	54.8%, (52.5, 57.1)

Bed nets that were Campaign bed nets	33.4%, (31.4, 35.4)

Bed nets that were Hanging	67.1%, (65.3, 69.1)

Bed nets in Good Condition	88.4%, (87.0, 89.8)

N = 3141 bed nets of any type in survey

N = 2092 ITN/LLINs in survey

Among all bed nets, 66.2% were either treated in the last six months (ie, were ITNs) or were LLINs. More than half (54.8%) of all nets were LLINs. Among all bed nets, 43.1% were obtained at no cost. Nearly two-thirds (67.1%) of all nets were hanging. About a third (30.6%) of all nets were obtained at health facilities (other than as a part of the campaign), and 24.8% were obtained from dukas (small stores), hawkers, kiosks, or textile shops. Almost ninety percent (88.4%) of nets were classified as being in good condition (no holes, tears). For the most popular brand of socially marketed bed net (a non-LLIN), 30.6% had been treated in the last six months.

### Households

About two-thirds (66.9%) of all households had at least one bed net of any kind. Over forty percent (41.9%) of households had more than one bed net. Over half (50.7%) of households owned an ITN, and 39.1% of all households had received a campaign LLIN. ITNs were hanging in 41.9% of all households. In households owning ITNs, 82.6% had at least one ITN hanging. Households that owned only campaign nets were 13.8% of all households, which was over one-third of all households receiving campaign nets. Summary data with 95% confidence intervals are presented in Table 4.

**Table 4:** Survey estimates related to households (HH)*

	Percent, (95% Confidence Interval)
Owns at least one bed net	66.9%, (64.8, 69.0)

Owns > 1 bed net	41.9%, (39.7, 44.0)

Owns an ITN/LLIN	50.7%, (48.4, 52.9)

Owned campaign bed net	39.1%, (29.9, 48.1)

All nets were from campaign	13.8%, (12.3, 15.4)

Any ITN/LLIN ownership:	

Urban HH	57.5%, (51.6, 63.4)

Rural HH	49.7%, (47.2, 52.1)

Any ITN/LLIN ownership by Wealth Quintile:	

Poorest	50.5%, (45.6, 55.4)

Second Poorest	60.4%, (55.4, 65.4)
	

Mid Quintile	41.1%, (36.2, 46.0)

Second Richest	53.6%, (48.5, 58.7)

Richest	49.1%, (44.1, 54.2)

Any ITN/LLIN ownership by malaria transmission zone:	

Endemic	58.7%, (55.7, 61.7)

Epidemic (Rift)	48.4%, (44.1, 52.7)

Highlands/Seasonal	43.5%, (39.1, 47.9)

N = 2059 households in the survey

Households that had neither a CU5 nor a woman of reproductive age comprised 19.2% of all households. In this group, bed net ownership was 16.2% for any ITN, and 33% for any type of bed net. Urban households were more likely than their rural counterparts to have nets of any type. (ITNs: Odds Ratio: 1.37, 95% CI: 1.06, 1.77; Any Net: Odds Ratio: 2.35, 95% CI: 1.70, 2.35). Among all households that received campaign nets, 94.8% had retained all nets and 97.9% percent had retained at least one campaign net. There was no significant variation in retention by malaria transmission zone status.

### Children under five

There were 1,727 CU5s living in 1113 households in the survey. Approximately two-thirds (67.5% ) of households with CU5s, received campaign LLINs. The percent of these households receiving campaign LLINs did not vary significantly by region. Just under ninety percent (87.2%) of households with CU5s had a bed net of any type, with 74.4% owning an ITN. Summary data with 95% confidence intervals for households with CU5s are presented in Table 5.

**Table 5:** Survey estimates for Households (HH) with Children under 5 (CU5s)*

	Percent, (95% Confidence Interval)
Owns at least one Bed net	87.2%, (85.2, 89.3)

Owns an ITN/LLIN	74.4%, (71.8, 77.0)

Owns campaign bed net	67.5%, (64.6, 70.3)

Any ITN/LLIN ownership by Wealth Quintile of HH	


Poorest	67.2%, (61.1, 73.2)
Second Poorest	84.5%, (79.7, 89.4)

Mid Quintile	71.9%, (64.4, 79.5)

Second Richest	78.3%, (72.1, 84.5)

Richest	72.9%, (65.9, 79.9)

	

Owned Campaign bed nets by Wealth Quintile	

Poorest	61.6%, (55.9, 67.4)

Second Poorest	81.4%, (76.7, 86.2)

Mid Quintile	61.9%, (54.1, 69.7)

Second Richest	70.8%, (64.5, 77.1)

Richest	58.4%, (51.1, 65.7)

N = 1113 Households with Children Under 5.

Just over half (51.7%) of CU5s (95% CI: 48.8, 54.7%) slept under an ITN during the previous night. This proportion did not vary significantly by age or sex. There was significant variation by malaria zone/geographic region: endemic: 50.3%; epidemic: 41.4%, highland/seasonal 67.9% (p < .0001). In households with CU5s owning ITNs, 69.0% of children slept under them the previous night, and in households with CU5s owning ITNs that were hanging, 83.5% of children slept under them the previous night. Having received a campaign bed net greatly increased the likelihood that a child under 5 slept under an ITN the previous night (67.7 vs. 28.8%, p < .0001). Overall, 56.5% of the ITNs used by CU5s were campaign nets. Summary data with 95% confidence intervals for CU5s are presented in Table 6.

**Table 6:** Survey estimates for Children under 5 (CU5s)*

Variable	Percent,
	(95% Confidence Interval)
Percent CU5s sleeping under ITN previous night	51.7, (48.8, 54.7)
	

In HHs with ITN: Percent CU5s sleeping under ITN previous night	69.0, (65.9, 72.1)

In HHs with ITNhanging: Percent CU5s sleeping under ITN previous night	83.5, (81.0, 86.0)

	

Slept Under ITN previous night:	

Urban	58.6, (50.5, 66.8)

Rural	51.1, (47.9, 54.3)

	

Slept Under ITN previous night: Malaria Region:	

Endemic	50.3, (46.3, 54.3)

Epidemic	41.4, (35.7, 47.1)

Highland/seasonal	67.9, (62.3, 74.5)

	

CU5s receiving Campaign Nets by Wealth Quintile of HH:	

Poorest	48.6, (43.0, 54.1)

Second Poorest	70.9, (65.4, 76.3)

Mid Quintile	59.2, (51.4, 67.0)

Second Richest	63.6, (56.8, 70.3)

Richest	53.1, (45.9, 60.4)

N = 1727 Children under 5 in the survey.

### Women of reproductive age

There were 2,053 women of reproductive age (15-49 years, WORAs) living in 1,613 households. There were 182 pregnant women living in 180 households in the survey. Among pregnant women, 58.4% slept under a net of any type during the previous night (95% CI: 50.9, 65.9%); however, only 36.3% of pregnant women slept under an ITN. (95% CI: 29.0, 43.7%). Among all WORAs, 33.0% slept under an ITN during the previous night (95% CI: 30.6, 35.4%). In households that owned one or more ITNs, 55.9% of women of reproductive age slept under an ITN during the previous night. For pregnant women, this number was 56.7%. Summary data for WORAs with 95% confidence intervals are presented in Table 7.

**Table 7:** Survey estimates for Women of Reproductive Age (WORAs)*

Variable	Percent, (95% Confidence Interval)
Percent WORAs sleeping under ITN previous night	33.0, (30.6, 35.4)

In HHs with ITN: Percent WORAs sleeping under ITN previous night	55.9, (52.7, 59.1)

In HHs with ITN hanging: Percent WORAs sleeping under ITN previous night	67.5, (64.2, 70.8)

	

WORAs in HH receiving campaign bed nets	46.0%, (43.3, 48.8)

	

Slept Under ITN previous night:	

Urban	47.3, (40.7, 53.9)

Rural	31.2, (28.8, 33.7)

	

Slept Under ITN previous night: Malaria Region:	

Endemic	35.5, (32.2, 38.8)

Epidemic	30.1, (25.9, 34.4)

Highland/seasonal	32.4, (27.6, 37.2)

	

WORAs Sleeping under ITN/LLIN previous night	

Poorest	28.2, (23.3, 33.1)

Second Poorest	33.3, (28.1, 38.4)

Mid Quintile	31.2, (25.8, 36.6)

Second Richest	35.5, (30.3, 40.7)

Richest	35.6, (30.1, 41.0)

N = 2053 Women of Reproductive age in the survey.

## Discussion

The distribution of 3.4 million LLINs dramatically increased ownership, and reached two-thirds of the target households with the population most vulnerable to malaria-related morbidity and mortality. At the time of the campaign, this distribution represented the largest distribution of bed nets ever. While the distribution of bed nets in Ethiopia, undertaken later, was considerably larger (approximately 20.5 million bed nets), [18], the Kenya campaign remains the second largest.

Since 2002, Kenya had an effort to socially market subsidized bed nets [19]. This multi-year programme distributed 5.0 million bed nets vs. the 3.4 million bed nets distributed as part of the campaign. Prior to the campaign, 68.7% of all bed nets were socially marketed. Afterwards, socially marketed bed nets still represented 46.0% of all bed nets owned. The vast majority of these bed nets were sold prior to the development of LLINs. A possible concern has been that the users of socially marketed bed nets might be different than the user of bed nets that are distributed free to a targeted group. Noor *et al *[20] notes that there is greater coverage of infants in areas where nets were received by free distributions than by social marketing.

When interpreting the impact of the campaign on bed net ownership for all households in the survey, one must remember that only 51.7% had a CU5 and were, therefore, eligible to receive a campaign LLITN. Additionally, over one third of households with women of reproductive age did not have a CU5 in the household. Households with these women, who might potentially become pregnant, were not eligible to receive campaign nets, thus not addressing the cohort of newborns that arrive following the conclusion of a campaign. A number of strategies are thus needed to achieve bed net usage and ownership targets. In addition to campaigns, other important approaches include distribution of ITNs via antenatal clinics as an efficient way to reach pregnant women. In the 2003 Kenya DHS, 88% of pregnant women received antenatal care. Therefore, increasing ITN ownership/usage in this population towards targets via further targeted distributions is achievable.

Within the targeted population (CU5s), the campaign was highly successful. The survey found that 74.4% of households with CU5s now own an ITN, and that 87.2% own a bed net of any type. This would also represent ITN ownership if all of these are treated (only approximately 30% were treated within the past six months at the time of the survey). However, due to logistics and costs of re-dipping, future net distributions will concentrate on simply replacing nets that require retreatment with LLINs. A mass distribution every three years supplemented by those available through health facilities would seem to be the simplest way to keep large numbers of vulnerable populations well supplied with viable LLINs

The survey results demonstrated that retention of campaign bed nets was very high (95% of all households retained all campaign nets). Therefore, few campaign bed nets were being resold or given away. This finding has been replicated elsewhere and has remained the case in later follow-up surveys [16].

Despite the timing of the survey, usage figures were much higher than previous surveys (51.7% of CU5s slept under an ITN vs. 4.6% CU5s under any net the previous night in 2003) [17]. In homes with ITNs, the usage rates were even higher (69.0%) and higher still in homes where ITNs were hanging (83.5%) Finally, in perhaps the best measure of the impact of the campaign, CU5s in homes receiving campaign bed nets were approximately 40% more likely to sleep under an ITN during the previous night (67.7 vs. 28.8%, p <.0001). Nonetheless, the survey found that a substantial number of bed nets were not being hung or used. It is notable that households in the poorest wealth quintile were 59% more likely to receive campaign bed nets, but that target populations in the wealthiest quintile were still 20% or more likely than those in the poorest quintile to actually have slept under an ITN during the previous evening (Figure 3). Behavioural change and education programmes could help close the gap between ownership and usage. These efforts will be a vital part of any strategy to meet bed net ownership and usage targets [20,21].

## Conclusion

The campaign successfully increased ITN ownership and usage in targeted vulnerable populations. The survey identified the need for educational and behavioral change campaigns to promote proper usage by vulnerable populations. Finally, the survey quantified the populations of interest not covered by the targeted distribution and for some of these populations, and helped delineate obvious next steps for increasing LLINs ownership.

Future efforts should target getting bed nets to the remaining populations that still do not have them, address eventual replacement of regular ITNs with LLINs, and encourage proper use of the bed nets.

## Competing interests

The authors declare that they have no competing interests.

## Authors' contributions

AH drafted the protocol, helped analyse the data, and wrote the manuscript. RK assisted with the protocol and provided scientific direction for the bed net campaign and the survey. AM provided logistical support for the bed net campaign, and helped with protocol development. AW provided data collection and analysis support, JE provided data collection and analysis support, MH provided scientific assistance on the protocol and the manuscript. AN provided assistance in the mapping and sampling process as well as the manuscript. SS directed the survey and provided input for both the protocol and manuscript. RB provided sampling expertise and data collection assistance. JV provided scientific advice and helped write the protocol and manuscript. KL provided assistance in the protocol and manuscript writing. LS helped write the manuscript and provided assistance for the survey. WA helped write the protocol and provided logistical support for both the bed net campaign and survey.

All authors read and approved the final manuscript.
